# Venous thromboembolism during preoperative chemotherapy in the CRITICS gastric cancer trial

**DOI:** 10.1002/cam4.3118

**Published:** 2020-07-31

**Authors:** Astrid E. Slagter, Karolina Sikorska, Cecile Grootscholten, Hanneke W. M. van Laarhoven, Pehr Lind, Marianne Nordsmark, Elma Meershoek‐Klein Kranenbarg, Cornelis J. H. van de Velde, Nicole C. T. van Grieken, Johanna W. van Sandick, Edwin P. M. Jansen, Marcel Verheij, Annemieke Cats

**Affiliations:** ^1^ Department of Radiation Oncology Netherlands Cancer Institute Amsterdam The Netherlands; ^2^ Department of Biometrics Netherlands Cancer Institute Amsterdam The Netherlands; ^3^ Department of Gastrointestinal Oncology Netherlands Cancer Institute Amsterdam The Netherlands; ^4^ Department of Medical Oncology Cancer Center Amsterdam Amsterdam University Medical Center The Netherlands; ^5^ Department of Oncology Stockholm Söder Hospital Stockholm Sweden; ^6^ Karolinska Intitutet Stockholm Sweden; ^7^ Department of Oncology Aarhus University Hospital Aarhus Denmark; ^8^ Department of Surgical Oncology Leiden University Medical Center Leiden The Netherlands; ^9^ Department of Pathology Amsterdam University Medical Center Amsterdam The Netherlands; ^10^ Department of Surgical Oncology Netherlands Cancer Institute Amsterdam The Netherlands; ^11^ Department of Radiation Oncology Radboud University Medical Center Nijmegen The Netherlands

**Keywords:** cancer‐associated thrombosis, gastrectomy, gastric cancer, preoperative chemotherapy, venous thromboembolism

## Abstract

**Background:**

The occurrence of a venous thromboembolism (VTE) is common in patients with cancer. Gastric cancer has been associated with one of the highest risks for VTE. Chemotherapy, especially cisplatin has been associated with a high VTE risk. In this study, risk factors for VTE occurrence and their potential impact on subsequent therapeutic interventions were investigated in patients who underwent preoperative chemotherapy, in the CRITICS gastric cancer trial.

**Patients and methods:**

Patients with resectable gastric cancer were preoperatively treated with three cycles of 3‐weekly epirubicin, cisplatin or oxaliplatin, and capecitabine (ECC/EOC). VTE was defined as any thrombus in the venous system, excluding superficial and/or device related VTEs. Potential risk factors were analyzed in a multivariable regression model with age, gender, Body Mass Index (BMI), tumor localization, Lauren classification, type of chemotherapy (ECC/EOC), (cardiovascular) comorbidity, and previous VTE as independent risk factors. The impact of VTE on completion rate of preoperative chemotherapy, surgical resection rate, postoperative complications, and start of postoperative therapy were investigated.

**Results:**

Of 781 patients, 78 (10%) of 781 patients developed a VTE during preoperative chemotherapy. On multivariable analysis, BMI ≥ 30 kg/m^2^ and previous VTE were associated with VTE occurrence (reference BMI < 25 kg/m^2^; OR 2.190; 95% CI 1.152‐4.164; *P* = .017/previous VTE; OR 3.617; 95% CI 1.201‐10.890; *P* = .022). Treatment with cisplatin was, compared to oxaliplatin, not significantly associated with VTE occurrence (OR 1.535; 95% CI 0.761‐3.094; *P* = .231). VTE occurrence did not affect completion of preoperative chemotherapy, surgical resection rate, postoperative complications, or start of postoperative therapy.

**Conclusion:**

High BMI and previous VTE were independent risk factors for VTE occurrence during preoperative chemotherapy in patients with resectable gastric cancer. VTE occurrence in the preoperative setting did not affect receipt of further treatment.

## Graphical abstract

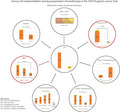

**Key messages**

- High BMI and previous VTE were independent risk factors for VTE occurrence during preoperative chemotherapy in patients with resectable gastric cancer.
- Comparable proportions of patients proceeded to surgery and started postoperative therapy, suggesting that VTE occurrence during preoperative treatment did not affect receipt of further treatment.

## 1 INTRODUCTION

The occurrence of a venous thromboembolism (VTE) is common in patients with cancer.^1^ The risk for VTE varies among different cancer subtypes, with gastric cancer having one of the highest risks.^2^ A VTE incidence of 16% has been described for patients with gastric cancer within 12 months after the start of chemotherapy.^3^ The underlying mechanism for this phenomenon is not completely understood. A combination of factors involved in the so‐called Virchow's triad may play a role in its pathogenesis: that is, blood stasis, vascular injury, and blood hypercoagulability.^4^

The development of VTE in patients with gastric cancer has been associated with clinical characteristics, such as age, gender, Body Mass Index (BMI), primary tumor localization, stage, grade/histology, major surgery and treatment with chemotherapy.^5^, ^6^, ^7^ For several of these associations an interaction with both the vascular system and coagulation cascade has been hypothesized as the underlying mechanism. Obesity‐driven chronic inflammation and hypofibrinolysis may induce a prothrombotic state.^8^, ^9^ Platinum‐based chemotherapy induces endothelial cell damage via formation of procoagulant endothelial microparticles, causing platinum‐related hypercoagulability.^10^ Within the group of platinum derivatives, cisplatin has been associated with a higher VTE risk than oxaliplatin.^11^, ^12^

As effective preventive measures, such as anticoagulant therapy, are available, early identification of high‐risk patients is warranted in order to reduce VTE‐related morbidity and mortality, hospitalization, and consequent costs.^13^, ^14^ Studies investigating risk factors and clinical consequences of VTE in patients with gastric cancer have mainly been carried out in Asia and/or in patients with metastatic disease. Differences in VTE incidence have been reported between ethnicities, with Asian cancer patients having a lower incidence compared to Caucasian or African‐American patients.^2^

In the current study, we investigated risk factors for VTE in the preoperative chemotherapy setting and their potential consequences for further therapy in Western patients with resectable gastric cancer treated in the CRITICS trial.^15^

## 2 METHODS

For the present study, we used data from the CRITICS trial, which enrolled patients with stage Ib‐IVa histologically proven adenocarcinoma of the stomach or esophagogastric junction with the tumor bulk in the stomach.^15^

All patients were randomized upfront to receive preoperative chemotherapy followed by surgery. They then either continued with postoperative chemotherapy or postoperative chemoradiotherapy. Preoperative chemotherapy consisted of three cycles of 3 weekly epirubicin (50 mg/m^2^ on day 1), cisplatin (60 mg/m^2^ on day 1), or oxaliplatin (130 mg/m^2^ on day 1), and capecitabine (either 1000 mg/m^2^ twice daily on day 1‐14 in combination with epirubicin and cisplatin or 625 mg/m^2^ twice daily on day 1‐21 in combination with epirubicin and oxaliplatin) (ECC/EOC). The ECC combination was the treatment of choice in the Netherlands and Denmark, whereas the EOC combination was the preferred treatment in Sweden. The CRITICS protocol specified that time between the last chemotherapy cycle and surgery was 3‐6 weeks.

Adverse events were prospectively collected from the moment of randomization using the common terminology criteria for adverse events (CTCAE) version 3.0. Detection of VTE was based on symptoms or clinical signs and/or during (predefined) radiological examinations. In this study, VTE was defined as any thrombus in the venous system, excluding superficial and/or device related VTEs. The preoperative treatment period was defined as time between start of preoperative treatment until the end of the last cycle plus 30 days, or day of surgery, whichever occurred first. Median time until VTE occurrence was defined as time between the first preoperative cycle until detection of VTE. In cases where more than one VTE occurred during preoperative treatment, the first diagnosed VTE was selected.

Baseline characteristics were scored at the time of inclusion and were compared between patients who developed a VTE and those who did not. Furthermore, we investigated whether VTE occurrence influenced the type of surgery (curative or palliative), or postoperative complications and whether or not patients started postoperative therapy (after curative surgery). Postoperative complications (after curative or palliative surgery) were categorized as general (cardiovascular, pulmonary, renal, and neurological), infectious (abdominal wound, abscess, and sepsis), and surgical complications (bleeding, anastomotic leakage, abdominal wound dehiscence, ileus, and intestinal necrosis).^15^

### 2.1 Statistical analysis

Statistical analysis was performed using SPSS statistical software, version 25. Categorical outcomes are presented as frequencies and percentages. Continuous variables are presented as medians and interquartile ranges (IQR). The association between VTE and age was tested using the Mann‐Whitney *U* test. The association between VTE and gender, tumor localization, type of chemotherapy (ECC or EOC), comorbidity, cardiovascular comorbidity and previous VTE were tested using the chi‐square test, or, if the number of patients was ≤10 (in one of the cells) with the Fisher's exact test. The association between VTE and BMI category was tested using linear‐by‐linear association test. The association between possible risk factors at baseline, and VTE was further explored in a multivariable logistic regression analysis. The threshold for statistical significance was set at 0.05.

## 3 RESULTS

A total of 781 patients started preoperative chemotherapy, of whom 78 (10%) developed a VTE in the preoperative treatment period. None of the patients developed a VTE between randomization and start of treatment. The median time until the occurrence of a VTE from the start of preoperative treatment was 6 weeks (IQR 5‐8 weeks). The first occurrence of a VTE was pulmonary embolism (PE) in 37 (47%) patients, deep venous thrombosis (DVT) in one of the extremities in 31 (40%) patients, both DVT and PE in one (1%) patient, thrombosis at another localization in 8 (10%) patients and not otherwise specified in one (1%) patient. Other localizations included the atrium, portal vein, renal vein, vena cava/ovarian vein, jugular vein, cerebral sinus thrombosis, and mesenteric vein/ root.

Among patients with a high BMI (30 kg/m^2^ or higher, n = 105), 17 (16%) patients developed a VTE during preoperative chemotherapy. Among patients with a VTE in their medical history (n = 17), 5 patients (29%) developed a VTE during preoperative chemotherapy.

The characteristics of patients who did and who did not develop a VTE during preoperative chemotherapy are shown in Table 1. In the same table, results of the univariable and multivariable analyses are presented. A BMI of 30 kg/m^2^ or higher was independently associated with VTE occurrence (OR 2.190; 95% CI 1.152‐4.164; *P* = .017). In addition, we identified previous VTE as an independent risk factor for development of a VTE during preoperative chemotherapy (OR 3.617; 95% CI 1.201‐10.890; *P* = .022). The other investigated baseline characteristics (age, gender, tumor localization, Lauren classification, and type of chemotherapy) were not significantly different for patients who did and who did not develop a VTE during preoperative chemotherapy.

**Table 1:** Baseline characteristics, univariable and multivariable analysis (^a^ reference variable)

Variable	Univariable analysis			Multivariable analysis		
	No VTE (n = 703)	VTE (n = 78)	*P* value	OR	95% CI	*P* value
Age in years (median; IQR)	62 (54‐69)	62 (56‐68)	.874	1.002	0.978‐1.027	.856
Sex			.412			
Male	474 (91%)	49 (9%)		1^a^		
Female	229 (89%)	29 (11%)		1.304	0.782‐2.174	.309
BMI			.022			
<25	369 (92%)	33 (8%)		1^a^		
25‐30	246 (90%)	28 (10%)		1.293	0.751‐2.226	.354
≥30	88 (84%)	17 (16%)		2.190	1.152‐4.164	.017
Tumor localization			.433			
EGJ	119 (89.5%)	14 (10.5%)		1^a^		
Proximal	156 (93%)	12 (7%)		0.657	0.288‐1.499	.318
Middle	187 (89.5%)	22 (10.5%)		0.975	0.466‐2.040	.947
Distal	238 (89%)	29 (11%)		1.065	0.522‐2.173	.863
Entire stomach	3 (75%)	1 (25%)		4.164	0.378‐45.836	.244
Lauren classification			.449			
Intestinal	221 (88%)	31 (12%)		1^a^		
Diffuse	213 (92%)	19 (8%)		0.639	0.340‐1.201	.164
Mixed	40 (89%)	5 (11%)		0.975	0.353‐2.694	.960
Unknown	229 (91%)	23 (9%)		0.739	0.411‐1.328	.311
Type of chemotherapy			.171			
EOC	139 (93%)	10 (7%)		1^a^		
ECC	564 (89%)	68 (11%)		1.535	0.761‐3.094	.231
Any comorbidity			.358			
No	372 (90.5%)	37 (9.5%)		^b^		
Yes	331 (89%)	41 (11%)				
Cardiovascular comorbidity			.635			
No	434 (90%)	46 (10%)		^b^		
Yes	269 (89%)	32 (11%)				
Previous VTE			.021			
No	691 (90%)	73 (10%)		1^a^		
Yes	12 (71%)	5 (29%)		3.617	1.201‐10.890	.022

Note

Percentages in this table represent the proportion of patients who did and did not develop a VTE during preoperative chemotherapy.

Abbreviations: BMI, body mass index in kg/m^2^; ECC, epirubicin + cisplatin + capecitabine; EGJ, esophagogastric junction; EOC, epirubicin + oxaliplatin + capecitabine; IQR, interquartile range; VTE, venous thromboembolism.

^b^ The categories “any comorbidity” and “cardiovascular comorbidity” were removed from the multivariable analysis because of multicollinearity.

A VTE occurrence during preoperative chemotherapy did not affect the proportion of patients who completed preoperative chemotherapy (three cycles): 590 (84%) patients without VTE and 65 (83%) patients with VTE completed three preoperative cycles of chemotherapy (*P* = .893).

The decision to proceed to surgery was not influenced by the diagnosis of a VTE during preoperative treatment. A total of 666 (95%) patients without VTE continued to surgery compared to 74 (95%) patients with a VTE (*P* = .960). Among the intention to treat population the operation was potentially curative in 575 (82%) patients without VTE and in 61 (78%) patients who developed a VTE during preoperative chemotherapy (*P* = .263).

Postoperative infectious complications were slightly more common in patients with VTE compared to patients without VTE (30% vs 20%, *P* = .058), which did not reach statistical significance. General postoperative complications and surgery‐related complications were comparable for patients without vs patients with VTE, 177 (27%) vs 16 (22%) patients (*P* = .325) and 137 (21%) vs 16 (22%) patients (*P* = .877), respectively. The amount of blood loss, in particular, was not affected by the occurrence of a VTE during preoperative chemotherapy. Median blood loss during surgery was 350 (IQR 200‐650) mL in patients without VTE and 400 (IQR 137.5‐700) mL in those with VTE (*P* = .704). Postoperative mortality was similar in both groups: 2% (n = 16) in the group of patients without VTE and 3% (n = 2) in the group of patients with VTE (*P* = .699). More details on the postoperative course are shown in Table 2.

**Table 2:** Postoperative complications in patients who underwent surgery (both curative and palliative surgery)

Variable	No VTE (n = 666)	VTE (n = 74)	*P* value
Infectious complications^a^	Missing n = 9		.058
Yes	133 (20%)	22 (30%)	
No	524 (80%)	52 (70%)	
General complications^b^	Missing n = 9		.325
Yes	177 (27%)	16 (22%)	
No	480 (73%)	58 (78%)	
Surgery‐related complications^c^	Missing n = 9		.877
Yes	137 (21%)	16 (22%)	
No	520 (79%)	58 (78%)	
Median (IQR) amount of blood loss (mL)	Missing n = 108	Missing n = 12	.704
	350 (200‐650)	400 (138‐700)	
In hospital mortality			.699
Yes	16 (2%)	2 (3%)	
No	650 (98%)	72 (97%)	

^a^ Including abdominal wound, abscess, and sepsis.

^b^ Including cardiovascular, pulmonary, renal, and neurological complications.

^c^ Including bleeding, anastomotic leakage, abdominal wound dehiscence, ileus, and intestinal necrosis.

Finally, VTE development during preoperative chemotherapy did not affect the administration of postoperative treatment, that is, 436 (76%) patients without VTE compared to 42 (69%) with a VTE started postoperative therapy (*P* = .231).

## 4 DISCUSSION

The current analysis was performed to identify risk factors for VTE occurrence in patients with resectable gastric cancer during preoperative chemotherapy and to investigate the potential consequences on treatment continuation. To the best of our knowledge, this is the first study investigating risk factors for VTE during (preoperative) chemotherapy in Western patients with resectable gastric cancer.

In this study, 10% of the patients developed a VTE during preoperative chemotherapy. This percentage is in line with other studies, performed in patients treated with preoperative chemo(radio)therapy for resectable esophagogastric cancer, reporting incidences between 3% and 14%.^16^, ^17^, ^18^, ^19^ In the current analysis, a BMI of 30 kg/m^2^ or higher and previous VTE were identified as independent risk factors for VTE occurrence during preoperative chemotherapy for resectable gastric cancer. A BMI of 30 kg/m^2^ or higher doubled the risk of VTE development in our study. Two other (Japanese) studies identified a high BMI (25 kg/m^2^ or higher) as a risk factor for VTE in patients with gastric cancer.^6^, ^20^

In the general population without cancer, obesity has been associated with a higher incidence of VTE. In a retrospective study in over 12 million patients, those with a clinical label of “obesity” had a twofold increased risk of developing VTE.^21^ A Dutch case‐control study confirmed a twofold increased risk for VTE development in obese patients compared to nonobese patients.^22^ In obese patients, higher plasma levels of coagulation factors VIII and IX were found. However, the effect of obesity did not change after adjustment for coagulation factor levels, suggesting that other factors present in patients with obesity may contribute to the risk of VTE development.^22^ Obesity is known to induce a low‐grade status of inflammation.^8^, ^9^, ^23^ Inflammatory cells may activate procoagulant factors, other than factors such as VIII and IX, and may lead to VTE development.^8^, ^9^, ^24^ Furthermore, obesity leads to higher abdominal pressure with consequently increased venous stasis and reduced forward flow.^25^

It is well‐known that patients with a previous VTE are at risk for VTE recurrence. A population‐based study showed that the risk for recurrent VTE is higher in patients with cancer than in patients without malignancies: 30/100 patient‐years for patients with cancer compared to 12.8/100 patient‐years for patients without cancer. The risk of recurrent VTE correlated with the extent of cancer spread.^14^ In our study we established that patients with gastric cancer had a higher risk for developing a VTE during preoperative chemotherapy if they previously had experienced a VTE, compared to patients who did not. Patients with a previous VTE had a threefold higher risk of developing a VTE during preoperative chemotherapy, with an absolute incidence of 29%.

A meta‐analysis including over 8.000 patients with different types of advanced solid cancers reported a higher incidence of VTE in cisplatin‐treated vs noncisplatin‐treated patients (1.92% and 0.79%, respectively).^26^ In a 2×2 randomized trial, including 1002 patients with advanced esophagogastric cancer, the VTE incidence was higher in patients receiving a cisplatin‐containing chemotherapy combination (15.1%), than in patients who received an oxaliplatin‐containing chemotherapy combination (7.5%).^12^ In our study, there was no significant relationship between cisplatin or oxaliplatin and VTE occurrence. This could be explained by a less prominent relationship between cisplatin and VTE in patients with localized gastric cancer than in patients with metastatic disease, for example, due to lower tumor load or due to other factors involving the Virchow's triad. However, it should be noted that this study was not powered to identify risk factors for VTE.

In patients with cancer, a diagnosis of VTE has been associated with decreased quality of life and high costs.^27^ Several studies investigated the role of thromboprophylaxis in patients with cancer. These studies included a variety of patient populations, and also the prophylactic measures were diverse.

In one of these studies, 1150 (unselected) patients with metastatic or locally advanced solid cancers were randomized between prophylactic nadroparin vs placebo.^28^ The incidence of VTE was significantly lower in the intervention (2.0%) group than in the placebo (3.9%) group, whereas both major and minor bleeding events were increased. In the CASSINI trial, 841 high‐risk patients with solid tumors (with or without metastases) were randomized between rivaroxaban or placebo, to evaluate whether rivaroxaban was safe and effective in VTE prevention.^29^ VTEs occurred in 6.0% of the intervention group, compared to 8.8% in the placebo group (*P* = .10). Body weight and previous VTE were not reported. Based on the current evidence, the role of thromboprophylaxis in patients with gastric cancer in the outpatient setting is still uncertain. Prospective randomized trials would be necessary to investigate the (cost‐) effectiveness and risks of thromboprophylaxis in patients with resectable gastric cancer.

The newly updated 2019 ASCO guidelines state that high‐risk outpatients may be offered thromboprophylaxis with apixaban, rivaroxaban, or a low‐molecular‐weight heparin during systemic chemotherapy, if there are no significant risk factors for bleeding and no potential drug interactions.^30^ Patients with a Khorana score of 2 or more are considered as high risk, indicating that every patient with gastric cancer is a high‐risk patient.^31^ Despite the fact that direct oral anticoagulants (DOACs) have shown a more favorable risk‐benefit profile compared to warfarin, use of a DOAC has been associated with a 25% increased risk of gastrointestinal bleeding.^32^ In the Hokusai VTE cancer trial, patients with gastrointestinal cancer had an increased risk of bleeding during treatment for recurrent VTE with endoxaban compared to patients treated with dalteparin.^33^ Although the updated ASCO guidelines do not mention the presence of gastrointestinal cancer as a contraindication for DOAC administration, a low‐molecular‐weight heparin (LMWH) may be more appropriate for these patients.

### 4.1 Strengths and limitations

To our knowledge, this is the first study investigating risk factors for VTE in Western patients with resectable gastric cancer. Our analysis was performed in a relatively homogeneous group of prospectively enrolled patients with primary, nonmetastatic resectable gastric cancer. Furthermore, the effect of developing a VTE during preoperative treatment on the ability to receive further treatment (surgery and postoperative therapy) was not investigated before. This study also has some limitations. First, it is a subanalysis of the CRITICS trial and was therefore not powered to identify risk factors for VTE. It is possible that we were not able to confirm certain risk factors due to the limited number of events (n = 78). Furthermore, we confirmed a significant association between having had a previous VTE and the risk of VTE occurrence during preoperative chemotherapy, but data on the use of anticoagulants at baseline were incomplete.

In conclusion, this study identifies patients with gastric cancer at high risk for VTE development. High BMI and previous VTE were identified as independent risk factors for VTE development during preoperative systemic chemotherapy. Randomized trials are warranted to investigate the role of thromboprophylaxis in patients with gastric cancer at high risk for VTE development.

## ETHICS

The CRITICS trial has been performed according to the declaration of Helsinki and received approval by the Medical Ethical Committee of the Netherlands Cancer Institute‐ Antoni van Leeuwenhoek. All included patients gave their informed consent prior to their inclusion in the CRITICS trial.

## CONFLICT OF INTEREST

HWM van Laarhoven reported receiving grants/medication support from Bayer, BMS, Celgene, Janssen, Lilly, Nordic Pharma, Philips, Roche, Servier, and serving on an advisory board for BMS, Lilly, MSD, Nordic Pharma, Novartis, Servier. NCT van Grieken reported receiving grants from the Dutch Cancer Society and The Netherlands Organization for Health Research and Development, and serving on an advisory board for Bristol‐Myers Squibb and Merck Sharp & Dohme. EPM Jansen, A. Cats and M. Verheij reported receiving grants from the Dutch Cancer Society, the Dutch Colorectal Cancer Group and Hoffmann La Roche. All remaining authors have declared no conflict of interest.

## AUTHOR CONTRIBUTIONS

AES, EPMJ, MV, and AC were involved in conception and design and drafting manuscript. All authors were involved in acquisition of data, interpretation of the data, revising manuscript, final approval of the manuscript, and accountable for all aspects of the work. AES and KS were involved in analysis of the data.

## Data Availability

Data that support the findings of this study are currently not publicly available.
